# Cooperative planning for physically interacting heterogeneous robots

**DOI:** 10.3389/frobt.2024.1172105

**Published:** 2024-03-13

**Authors:** Michael A. Sebok, Herbert G. Tanner

**Affiliations:** Department of Mechanical Engineering, University of Delaware, Newark, DE, United States

**Keywords:** heterogeneous multi-agent systems, robot planning and control, cooperative planning, hybrid automata, physical interaction

## Abstract

Heterogeneous multi-agent systems can be deployed to complete a variety of tasks, including some that are impossible using a single generic modality. This paper introduces an approach to solving the problem of cooperative behavior planning in small heterogeneous robot teams where members can both function independently as well as physically interact with each other in ways that give rise to additional functionality. This approach enables, for the first time, the cooperative completion of tasks that are infeasible when using any single modality from those agents comprising the team.

## 1 Introduction

While cooperative robot behavior for spatial deployment and object manipulation has been studied in length both for homogeneous and heterogeneous robot groups, the latter case can leverage both plurality and *diversity* to complete tasks that would be nontrivial or impossible with a homogeneous group of any single available robot modality. The possibility of exploiting robot heterogeneity and diversity coupled with *physical interaction* to carry out otherwise infeasible missions has not been adequately investigated, and as a result part of a collaborative system’s potential remains untapped. In the function of heterogeneous human teams, teammate diversity can be instrumental when leveraged properly in a variety of application scenarios. There are instances in literature where robot heterogeneity was essential in accomplishing the task at hand. Mellinger et al. (2013) use a combination of a wheeled mobile robot and a quadrotor to extend the operational range of the latter. Another example is the work of Kiener and von Stryk (2007) in which a robotic wheelchair needs to transport a humanoid to a location where the humanoid needs to kick a ball. Yet another example is the approach of Huntsberger et al. (2003) who consider multiple planetary rovers which may not necessarily differ morphologically, but they have different capabilities. In general, however, fully leveraging diversity and heterogeneity in heterogeneous robot teams is nontrivial. The development of efficient planning and control algorithms for heterogeneous groups presents unique challenges due to the varied capabilities and operational constraints of individual agents. What is more, the intricacies of nontrivial types of physical interaction necessitate the incorporation of some of the agents’ underlying continuous dynamics, which significantly complicates analysis and inhibits planning algorithm scalability.

Consider a scenario where a small heterogeneous robot team is tasked to complete a mission that is beyond the inherent capabilities of any of its members. Assume that the inherent capabilities of robot team members are enabled by appropriate underlying control loops. Each control loop gives rise to one of a finite collection of possible actions that the robot can carry out. The robots comprising this team are capable of physically interacting (e.g., pushing, pulling, lifting, etc.) with each other and possibly with portions of their environment.

**FIGURE 1:**
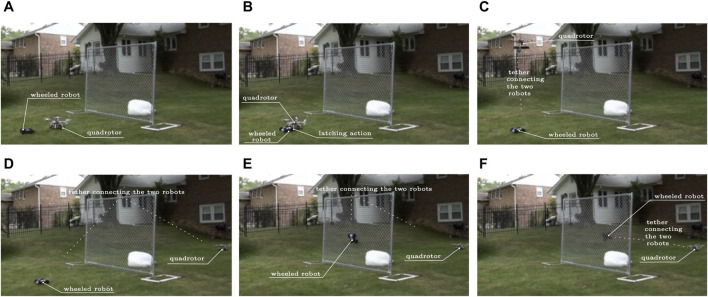
Cooperation of robots utilizing physical interaction for overcoming otherwise insurmountable obstacles. **(A)** The quadrotor lands in front of the ground vehicle; **(B)** The tip of the ground vehicle’s spool attaches to the quadrotor’s velcro apron; **(C)** The quadrotor takes off to fly over the fence, tethered on the ground vehicle which lets the line reel out; **(D)** The quadrotor lands on the other side of the fence; **(E)** The ground robot uses its powered spool to reel in the line and climb vertically against the fence; **(F)** The ground robot has made it over the fence and is on its way to the soft landing area on the other side of the fence.

Physical interaction between a robot and its environment has been demonstrated to be potentially advantageous for mission completion (Karydis et al., 2014; Stager and Tanner, 2020; Stager and Tanner, 2016; Stager and Tanner, 2019). In addition, some types of physical interaction between robots can significantly change the dynamics of the agents involved (e.g., when an aerial robot lifts a wheeled robot, the aerial robot’s inertial characteristics change and contact constraints for the ground robot are now lifted) (Mellinger et al., 2011). Here we are focusing on the latter kind of interaction, and are particularly interested in tasks that no single modality of robots in this team, regardless its scale, can carry out on their own (e.g., wheeled robots cannot jump to the other side of a fence; see Figure 1); meanwhile, a deliberate interaction and physical coupling between the heterogeneous teammates can make this task possible. This paper describes a methodological framework for identifying the type of physical interactions needed for the desired cooperative task completion, and for planning the sequence of actions that the robot teammates need to undertake to execute the task. It should be noted that physical interaction with the environment as well as non-physical interaction between agents are also allowable within this framework. However, non-physical interactions have been extensively studied within the existing literature so this work primarily focuses on the planning difficulties unique to physical interaction.

In the case of Figure 1, the sequence of cooperative actions enabling the ground robot to climb over the fence utilizing “togglable” physical interaction with an aerial vehicle through a powered spool mechanism was manually scripted, and the robots were remotely controlled. This paper offers a methodology for *automating* such planning and mission execution processes through an algorithmic framework focusing on a subclass of heterogeneous robot groups where physical interaction between teammates can transform the dynamics of the combined system. This application of physical interaction enables planned actions that are not within the action space of any agent acting individually. Existing planning and control theoretical frameworks for heterogeneous groups are poorly equipped to model system functionality that exists due to physical interaction and fully leverage this type of heterogeneity. This paper therefore (i) narrows the gap by providing methodological innovations in modeling cooperative behavior arising from nontrivial system coupling, and (ii) demonstrates that they facilitate the discovery of cooperative robot plans that are beyond the solution space of existing approaches.

To enable and regulate coupling between the cooperating robotic agents, as well as accurately predict agent evolution in their physical workspace, a suitable hybrid dynamical system modeling formulation is adapted to capture the full closed-loop dynamics of the robot team members. Then, a new formal operation for *composing system behavior* in heterogeneous robot teams, inspired by constructs found in the field of computational and mathematical linguistics, is introduced and exploited to create a comprehensive model of system dynamics and available behaviors. This (discrete) behavior composition approach offers two distinct advantages: it curbs the growth of computational complexity when robot teams increase in size, and it exposes new behaviors that become possible when individual team members couple with each other. Subsequently, discrete abstractions of the closed-loop hybrid agent dynamics are utilized to transfer the planning problem to a purely discrete space. When formulated in this way, and with guidance from appropriate objective functions that draw from the continuous system dynamics, discrete optimization algorithms can discover new solutions to cooperative planning problems that until recently would be considered infeasible.

This cooperative planning methodological framework is validated in two heterogeneous system case studies. The first is motivated by the scenario of Figure 1 and is also utilized in the remainder of this paper as a running example which strongly leverages physical interaction between individual robots. This study utilizes a pairing between an unmanned aerial vehicle (uav) and an unmanned ground vehicle (ugv) through a novel electromechanical design that allows optional and controlled uav–ugv tethering. The second case study leverages a different type of physical interaction with a wheeled ugv and a walking ugv that join together magnetically to give rise to a mobile manipulator.

## 2 Related work

### 2.1 Physical interaction in multi-agent systems

Physical interaction between robots is commonly used to extend the functionality of multi-robot systems. Tethered connections between robotic vehicles are only one example of how physical interaction can be realized and leveraged. In this particular direction, for instance, one can bring up the case of Miki et al. (2019), who feature a tethered uav/ugv system that can be used to climb obstacles and which bears conceptual similarities to the first case study treated in this paper. Others have used a tether simply to deliver additional power in a uav/ugv system (Ogusu et al., 2020; Sutera et al., 2020). Tethers were also used in a multi-ugv system to lower one of the vehicles over the edge of a cliff (Pirjanian et al., 2002) and a reversible connection (Casarez and Fearing, 2016) which allows two crawling vehicles to traverse a vertical step. Outside the tethered robotics area, we find the work of Mathews et al. (2010) who leverage a supervisory uav in coordinating a group of ugvs to connect together to form a bridge. The aforementioned cases are merely a small sample of examples in literature where physical interaction realized via mechanisms subject to complementarity constraints has been utilized in the context of cooperative multi-robot behavior. However, most of these attempts tend to focus primarily on the system dynamics of physical interaction *without* addressing the question of how such interaction can be utilized as a modality within a versatile high-level planning framework.

### 2.2 Optimal planning and control of hybrid systems

The simultaneous and coupled evolution of discrete and continuous dynamics featured in hybrid dynamical systems present enormous challenges to optimal control and planning (Cassandras et al., 2001; Egerstedt et al., 2006; Zhu and Antsaklis, 2015). In optimal control formulations like the ones mentioned, the sequence of discrete modes through which the system goes is typically *a priori* given; in this paper, this sequence is one of the key variables to be solved for. In general, existing methods for coordinating optimal behavior in heterogeneous (hybrid) multi-agent dynamical systems can generally be placed on a spectrum where on one end one finds purely discrete planners and on the other continuous optimization methods. One early example of the former for heterogeneous systems was proposed by Parker (2001) and enabled groups of ground vehicles to complete tasks such as cooperatively pushing and repositioning boxes. Fully discrete methods focus on optimizing over the set of discrete states while abstracting or approximating the dynamics of the agents on those states. Planners that work in the continuous space, on the other hand, can account for more complex dynamics but tend to struggle with hybrid mode switching, especially when the underlying system model changes drastically in terms of its continuous, discrete, or hybrid behavior. One example of a continuous approach to hybrid system planning is that of Posa et al. (2014) which efficiently handles complementarity (contact) constraints without having to distinguish between different discrete modes and engage in any sort of combinatorial analysis. Another example of an optimal planning and control method for hybrid systems that lies in between the two ends of the spectrum and could be applicable in the context of problems addressed in this paper is the hybrid optimal control method of Zhao et al. (2020). It is capable of handling both discrete and continuous dynamics, albeit of relatively small dimensions and with the latter having to be expressed in polynomial form. Still, because of its capacity to integrate nonlinear continuous dynamics it maps closer to the continuous end of the spectrum of available planning algorithms. On the reciprocal end, one may note the grstaps algorithm (Messing et al., 2022), which is primarily discrete, employing a high-level abstraction of the agent dynamics to derive optimal plans in multi-agent systems. Discrete planning algorithms fare better for systems with numerous agents and tasks, whereas continuous planners are more capable of handling continuous dynamics and may have a better chance of producing feasible motion plans.

### 2.3 Existing limitations and proposed solution

Existing planners are challenged when called to plan for heterogeneous multi-robot systems that require leveraging physical interaction between their teammates to complete tasks. The contact mechanics and its impact on the component system dynamics place significant challenges to existing approaches, often leading to paradoxical solutions or failing to identify feasible ones (Johnson et al., 2016).

Continuous planners consistently fail to capture the emergent behaviors enabled by physically interacting agents. And while discrete planners are considerably more efficient and scale better with the number of agents involved compared to continuous ones, the often over-simplifying approximations of the continuous dynamics of the agents can either lead to infeasible plans or miss a whole range of solutions that are enabled explicitly by the continuous dynamics that have been abstracted away. Planners that combine discrete and continuous aspects of system behavior are still severely limited in the classes of system dynamics they can handle, the size of the overall system in terms of number of states, and the lack of guarantees for finding feasible solutions.

The novel planner described in this paper attempts to retain some of the computational efficiency of discrete planners while taking the continuous dynamics of each location in the hybrid automaton into account—the caveat here, and the feature that facilitates more dynamically consistent abstractions for subsequent discrete planning, is the closure of control loops at the continuous layer.

## 3 Hybrid automata for cooperative behavior

One commonly employed modeling formalism for modeling an individual member of a multi-agent system is the *deterministic finite automaton*. However, such an automaton is a purely discrete model of computation and as such has severe limitations with respect to capturing the physical dynamics of a robot. On the other hand, its simplicity allows for plans to be generated in an efficient manner. A finite automaton can be defined as follows:

Definition 1 A finite automaton is a tuple $A=\left\langle L,A,L_{0},F,\Delta \right\rangle$ comprised of

**Table:** 

*L*	a finite set of locations;^(i)^
*A*	a finite set of labels;^(ii)^
*L*_0_	a finite set of initial (starting) locations
*F*	a finite set of final (end) locations;^(iii)^
Δ	the transition function.^(iv)^

(i) the discrete states of the automaton;
(ii) the alphabet of (input) symbols that trigger transitions between discrete states and thus label the edges of the automaton graph that represents the discrete behavior of the system;
(iii) a subset of *L* on which when the automaton is, it is said that it has accepted a finite string made of labels in *A*;
(iv) a function Δ: *L* × *A* → *L* which determines the new state of the automaton based on the current state and input label.

There is a particular discrete operation on *finite* automata that combines them in a way that yields an outcome system, the behavior of which is neither the intersection nor the union of the behaviors of its factors. In addition, the number of locations in the resulting automaton increases linearly (rather than exponentially) with respect to the number of locations of the components. The motivation for such an operation is found in its utility for language identification in the limit in the context of mathematical linguistics (Heinz and Rogers, 2013). In this paper, the idea behind this mechanism will find a new application in the domain of robotic planning. Let us first introduce the original construction:

Definition 2 (cf. Heinz and Rogers, 2013). Given two finite automata $A_{1}=\left\langle L_{1},A,L_{01},F_{1},\Delta_{1}\right\rangle$ , $A_{2}=\left\langle L_{2},A,L_{02},F_{2},\Delta_{2}\right\rangle$ , their *join* is a finite automaton $A_{1}\;⊔A_{2}\;=\;\left\langle L_{1}\;\cup L_{2},A,L_{01}\;\cup L_{02},F_{1}\;\cup F_{2},\Delta_{1}\;\cup \Delta_{2}\right\rangle$ .

**FIGURE 2:**
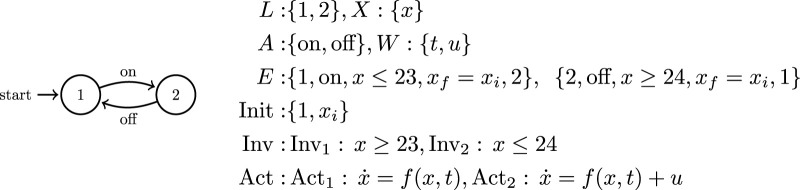
A standard wall thermostat as a typical example of a hybrid automaton: There are only two locations which correspond to the heater being either on or off. There is a single state variable, the indoor temperature *x* as well as two communication variables, the input heat *u* and the external temperature *t*. The hybrid automaton is constructed from these variables as a set of equations that govern system behavior and license discrete transitions between the two system locations.

This *join* operation enables the composition of individual automata representing each robotic agent in the multi-agent system into a single joined automaton which represents a comprehensive and efficient model of system capabilities. However, classical finite automata are not sufficient to model the complex dynamics that arise in some cooperative multi-agent systems and which must be considered to generate feasible planning solutions. To this end, the mathematical formulation in this paper leverages *hybrid automata* (Figure 2) and features a unique hybrid automata-based planning method. This method is computationally efficient and robust enough to compute plans for systems where physical interaction between agents is absolutely necessary for task completion. Hybrid system modeling formulations are attractive in this context because they allow one to capture salient features of the continuous robot dynamics that are important for task completion. They also offer appropriate mathematical handles to high-level discrete planners and optimizers. One of the multiple challenges in working with multi-agent systems in a hybrid framework is analytical *and* computational complexity. While the latter may be easy to see on the outset, part of the difficulty associated with the former is the need for incorporating (generally nonlinear) continuous dynamics as well as the possibility that the discrete structure of the overall system can experience changes during the course of the plan evolution. This paper builds on a particular modeling formalism for hybrid automata (van der Schaft and Schumacher, 2000):

Definition 3 A hybrid automaton consists of a tuple H=⟨L,X,A,W,E,Init,Inv,Act⟩ with components as follows:

**Table:** 

*L*	a finite set of locations;^(i)^
*X*	the continuous state space;^(ii)^
*A*	a finite set of labels;^(iii)^
*W*	the continuous communication space;^(iv)^
*E*	a finite set of events;^(v)^
Init	a set of initial states;^(vi)^
Inv	the invariants of continuous dynamics;^(vii)^
Act	activities, i.e., continuous vector fields;^(viii)^

(i) the nodes in the hybrid automaton graph;
(ii) a subset of $R^{n}$ where the system’s continuous variables belong to;
(iii) the discrete input alphabet that trigger transitions in the automaton’s graph and label its edges;
(iv) the domain of external variables that affect its behavior;
(v) the automaton’s transitions, as tuples of the form ⟨ initial location, label, guard condition, resets on continuous variables, new location ⟩;
(vi) a set of initial states (*ℓ*_0_, *x*_0_) ∈ *L* × *X* at which the hybrid automaton can be initiated;
(vii) the subsets of *X*, one for every *ℓ* ∈ *L* which remain invariant under the dynamics imposed by Act(*ℓ*).
(viii) a mapping of activities Act: *L* → *TX* which associates a continuous vector field in *TX* to each location in *L*.

In the way we utilize Definition 3 in this paper, we understand the invariants of locations as the limit sets (post conditions) of the activities labeled in the events leading to these locations, and the domains (pre conditions) of the activities of events that depart these locations. Note also that this hybrid model features no explicit (continuous) control inputs. Instead, we utilize the set *W* of communication variables to parameterize pre-defined feedback control action at each location, and pass on to the hybrid system relevant information about its environment and its teammates. The guard condition is key to determining the currently enabled transitions in the automata based on the current system state. We allow the guard conditions to be parameterized both by the continuous state space *X* as well as the communication variables *W*, a fact that aids in the construction of prerequisite conditions for collaborative action. We assume that the pre-defined continuous controllers in each location faithfully implement the high-level behavior prescribed by Act(*ℓ*), e.g., convergence to a desired region of the state space. One example of a hybrid system model is that of a wall thermostat controlling the heat within a home (Figure 2).

As written, Definition 2 is not suited for use with hybrid automata because it is constructed to operate on purely discrete systems wihtout any continuous dynamics. To address this issue, we have introduced Definition 3 to enable cooperative hybrid behavior. Thus, when heterogeneous systems are designed to interact with each other, their models reflect this capacity. One way of incorporating collaborative behavior is to include in each system (cooperative) events that can (only) be triggered by other systems. These events must be disabled within individual agent models so that they cannot be triggered by one agent acting alone. These modified events that result from collaborative actions are lacking a specified initial location, since they are only enabled in cooperation with another system which provides the initial location and matching label. The system, thus, could never trigger those events on its own, but the capacity is built into its model for the prospect of interaction with other systems. Labels “borrowed” from the label sets of other systems provide the framework for collaborative events that are only enabled when the requisite individual systems are joined together.

With this in mind, we implement the former blocking mechanism, which allows for events to incorporate labels external to the system. This is facilitated by expanding its label set to include the labels of the other components, i.e., setting *A* = *A*_1_ ∪ *A*_2_ as a common label set. This way, the component hybrid automata match the modeling specifications of the finite automata in Definition 2 (which share a common label set). The underlying assumption here is that $H_{1}$ and $H_{2}$ may not be able to utilize *every* label in *A* to trigger events; there could be some events that remain dormant when each system operates in isolation. Additionally, there may be certain locations within the individual hybrid automata that are essentially unreachable when operating in isolation as they might only be reachable under some sequence involving one of the aforementioned dormant events.

With the understanding that execution evolves in a *turn-based* manner, i.e., the component systems take turns executing actions and do not evolve concurrently—just as in the case of the finite automata join operation of Definition 2— we are now in position to extend the latter definition to hybrid dynamical systems:

Definition 4 Given two hybrid automata $H_{1}\;=\;\langle L_{1},L_{01},F_{1},X_{1},A,W_{1},$ $E_{1},{Init}_{1},{Inv}_{1},{Act}_{1}\rangle$ , $H_{2}\;=\;\left\langle L_{2},L_{02},F_{2},X_{2},A,W_{2},E_{2},{Init}_{2},{Inv}_{2},{Act}_{2}\right\rangle$ , their *join* is a hybrid automaton $H_{1}⊔H_{2}=\langle L_{1}\cup L_{2},L_{01}\cup L_{02},F_{1}\cup F_{2},$ $X_{1}\times X_{2},A,W_{1}\times W_{2},E_{1}\cup E_{2},{Init}_{1}\cup {Init}_{2},{Inv}_{1}\times {Inv}_{2},{Act}_{1}\times {Act}_{2}\rangle$ .

Note that the outcome of the operation the above definition does not strictly conform to the specifications of Definition 3 in the sense that Init_1_ ∪ Init_2_⊈(*L*_1_ ∪ *L*_2_) × (*X*_1_ × *X*_2_) but this difference is inconsequential given the turn-based evolution of the machine, and does not interfere with recursive applications of the operation. What is important is that with the union of locations and the union of events, the join system can now enable those previously dormant events. The mechanism of Definition 4 thus gives rise to a richer cooperative hybrid system behavior, which—as the colloquial saying goes—is “bigger than the sum of the parts.”

**FIGURE 3:**
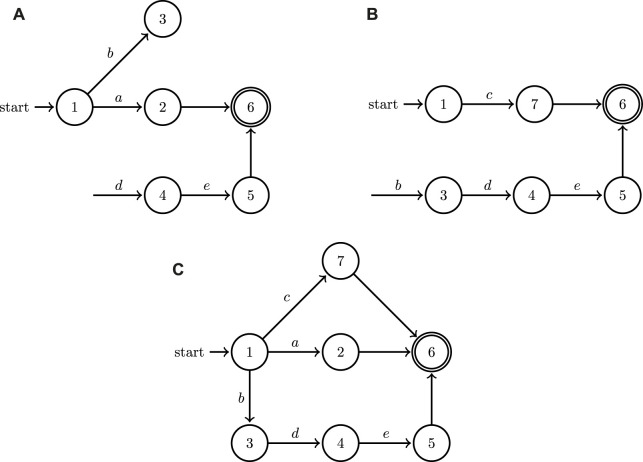
A minimal example of the application of the join operation to the graphs of two hybrid automata depicted in **(A,B)**; the outcome of the join operation is showcased in **(C)**. Both of the two factor automata share some input symbols (labels) and allow for discrete state resets.

Now that we have a suitable operation for capturing unique new cooperative behavior involving interacting hybrid dynamical systems through another hybrid automaton, the formalism can be leveraged to create models of multi-agent systems where cooperative interaction is essential to task completion. The (discrete) event dynamics of a hybrid automaton H can be visually represented in the form of a directed graph, the nodes and edges of which are labeled by *L* and *A*, respectively. In order to illustrate the functionality of the operation introduced in Definition 4, consider the automata in Figure 3. In Figure 3, events (transitions) that are not labeled express a decision on the part of the system to terminate execution at that point by transitioning to a final state. Notice in Figure 3 the mechanism for blocking cooperative behavior through event withholding when systems operate in isolation: location 4 (and consequently also 5) is unreachable in both systems; in system (A) the event associated to the transition from location 3 to location 4 is not included, and similarly in system (B) the event from 1 to 3 is missing. However, when the two systems join, their events combine and now both 4 and 5 can be reached through some collaborative action [first (A) moves to 3 with *b*, and then (B) moves to 4 with *d*].

This formulation, therefore, can capture both the behavior of individual systems when they operate in isolation as well as the new cooperative behaviors that become enabled when they are composed using the join operation under the turn-based assumption. Notice also that the outcome of the join operation [system (c) in Figure 3], is not significantly bigger compared to $H_{1}$ and $H_{2}$ . The size of this outcome scales *linearly* with the size of the location sets of its components.

While this paper only focuses on systems with two agents for representation brevity and clarity, Definition 4 directly extends recursively to any finite number of collaborative hybrid systems.

## 4 Cooperative plans with physical interaction

The modeling formulation of Section 3 captures the mechanics of deliberate physical interaction between heterogeneous robots and exposes cooperative plans that classical transition system composition operations ignore. Now, it is the job of an appropriately guided search algorithm to shift through the finite space of possible cooperative turn-based plans to single out the ones that allow the multi-agent system to achieve its objective.

It needs to be emphasized here that a naive approach utilizing a generic graph search is bound to fail: for example, the shortest path from initial to final locations in the join automaton of Figure 3C would be *a* or *c*, but that is not a valid solution to any task specification requiring collaborative action *e*. The search needs to be guided with information from the continuous dynamics of the hybrid automata and cannot be conducted using solely the discrete information contained in the automata graphs. This section outlines the implementation of efficient search methodology to find effective cooperative solution plans which can then be implemented by the agents in a heterogeneous system.

### 4.1 Discretization of the workspace

**FIGURE 4:**
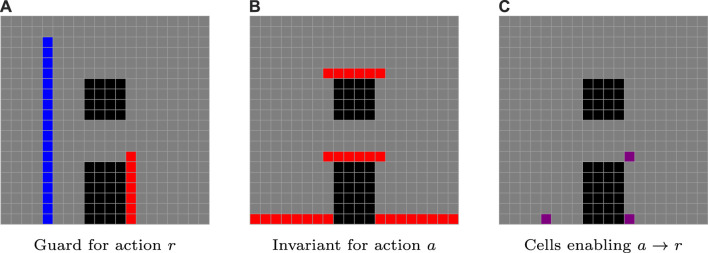
In this example drawn from the uav–ugv case study, analyzed in more detail in Section 5, a transition from a rolling activity *a* to a reeling activity *r* for a ugv is enabled at the intersection **(C)** of the guard of *r* **(A)** with the invariant of *a* **(B)**.

The first step in the planning process is to discretize the continuous shared robot workspace. This discretization is dictated by the structure of the guards and the invariants of the hybrid join automaton. Workspace cells produced by the discretization process are associated to locations of the join system and the assignment may not be one-to-one, i.e., cells may belong to multiple locations. This mapping of cells to locations results in each location having a set of cells assigned to its guard and another set assigned to its invariant. Informally, the guard set represents locations where a particular activity can be (forcibly) triggered (activities are linked to locations, the latter pointed to by labeled edges in Figure 3C). Similarly, the invariant represents the reachable space of the current activity (i.e., closed-loop dynamics). Cells associated with the same location that are mutually reachable using (possibly different parameterizations of) the low-level controller corresponding to a given activity can be grouped together into collections of cells subsequently referred to as *supercells.* Assume that the workspace is divided into *n* cells with individual cells denoted *q*_*i*_ with *i*: 1 → *n*. For each location *ℓ* ∈ *L* and associated label *a* ∈ *A*, supercells are constructed as a set of adjacent cells with overlapping faces (i.e., {*q*_*i*_, *q*_*j*_, … }) that all satisfy the continuous contraints of a particular invariant or guard. Each supercell associated with Inv(*ℓ*) is denoted $I_{\ell }^{r}=\left\{q_{i},q_{j},\ldots \right\}$ and each supercell associated with Guard(*a*) is denoted $G_{a}^{r}=\left\{q_{i},q_{j},\ldots \right\}$ with *ℓ* ∈ *L*, *a* ∈ *A* and *r*: 1 → *m* where *m* is the number of supercells associated with that invariant or guard. Constructed supercells are labeled with their corresponding action and an index since there can be several disjoint supercells within the guard or invariant of a particular location. Let the superset of all invariant supercells be denoted *S*_*I*_ and the superset of all guard supercells be denoted *S*_*G*_. Then, the individual invariant supercells collectively form set $S_{I}=\left\{I_{a}^{1},I_{a}^{2},I_{b}^{1},\ldots \right\}$ and the individual guard supercells form set $S_{G}=\left\{G_{a}^{1},G_{a}^{2},G_{b}^{1},\ldots \right\}$ . In this paper, the relationship between the set of supercells for the guards of and an action and the set for the invariants is one-to-one so that each guard always has an associated invariant. Individual cells are permitted to belong to multiple supercells corresponding to different locations of the hybrid automata, e.g., $q_{i}\in I_{a}^{1},q_{i}\in I_{b}^{2}$ . Exploiting this continuous state abstraction significantly reduces computation time when searching through the discretized workspace for an action sequence. For an event to be enabled and the corresponding transition to occur, the system must find itself at an intersection of the current invariant with the guard of the event’s destination location. The planner then optimizes over a sequence of labels (i.e., events) that take the system between different supercells, focusing on regions where a guard supercell for one location intersects with an invariant supercell of another location (see Figure 4).

### 4.2 The search for the right action sequence

A key input to our discrete planner is the set of cells where a transition between hybrid automaton locations is licensed. This happens when a supercell associated with the guard of one location intersects with a supercell associated with the invariant of another location. The set of transition cells for a pair of supercells $I_{a}^{i},G_{b}^{j}$ can be written as

$$Q_{I_{a}^{i}\to G_{b}^{j}}=\left\{q_{i}|q_{i}\in I_{a}^{i}\cap G_{b}^{j}\;\&\;\left\langle \ell_{i},\cdot ,\cdot ,\cdot ,\ell_{j}\right\rangle \in E\right\}$$

where *ℓ*_*i*_ is the location associated with supercell $I_{a}^{i}$ and *ℓ*_*j*_ is the location associated with $G_{b}^{j}$ . For every pairing of $I_{a}^{i}\in S_{I}$ and $G_{b}^{j}\in S_{G}$ such that $I_{a}^{i}\cap G_{b}^{j}\neq \emptyset$ , there exists one or more cells where a transition is enabled between the location associated with action *a* and the location associated with *b*. Only one of these transition cells is considered by the planner when calculating the cost to transition between the corresponding supercells. This “ideal” transition cell, denoted $q_{m}^{a_{i}\to b_{j}}$ , is the cell $q_{i}\in Q_{I_{a}^{i}\to G_{b}^{j}}$ which minimizes the future cost *h*_*j*_ evaluated at the cell centroids. If multiple cells fit this specification, one can be selected without significant impact on the nature of the final solution, since within the (landing) supercell individual cells are mutually reachable. Note that, before a transition between supercells occurs, the robot is located in cell $q_{m}\in I_{a}^{i}\cap G_{b}^{j}$ and after transition is located in the same cell within the invariant of the new supercell $q_{m}\in I_{b}^{j}$ until the next transition is enabled. With slight abuse of notation, to simplify notation in the description of our planner implementation, we represent the supercell transition $I_{a}^{i}\to G_{b}^{j}\to I_{b}^{j}$ as *ℓ*_*i*_ → *ℓ*_*j*_. With this in mind, we construct a matrix of transitions *T* consisting of tuples of the form {*ℓ*_*i*_, *ℓ*_*j*_, *q*_*m*_}, containing every pair of supercells *ℓ*_*i*_, *ℓ*_*j*_ licensed for transition, and the minimum cost *q*_*m*_ for that transition.

The planner developed for this problem (Algorithm 1) is a modified version of a forward A* algorithm that optimizes over the subset of cells where transitions between supercells can occur—instead of considering all the cells in the workspace. This algorithm is now informed of the (short-term) cost *a*_*ij*_ of reaching the transition cell between (super) cells *ℓ*_*i*_ and *ℓ*_*j*_ using a continuous function of the join hybrid system’s state. A design choice adopted in our implementation for quantifying the cost of such transitions is based on a particular type of scalar potential functions of the continuous state of the join hybrid automaton. These functions are known as *navigation functions* (Rimon and Koditschek, 1992) and they can serve as surrogates for a combined metric of “distance to goal” and “distance from constraint boundaries.” Just as in a typical A* algorithm, the planner tracks the running total cost *c*_*i*_ for each candidate sequence of transitions between supercells in a priority queue typically referred to as the open set. Additionally, there is a parameter termed upper , which tracks the cost of the current best sequence that reaches goal location *ℓ*_*f*_.

Algorithm 1 A* Planner with Supercells.

** Inputs:**
**  **
*T*, *M*
** Initialize:**
**  **

upper←∞

**  **
*c*_*m*_ ←*∞ ∀ ℓ* ∈ *M*
**  **

$open\leftarrow \left\{\ell_{1}\right\}$ *ℓ*_1_ is the initial location
** while** open≠∅ **do**
**  **Select sequence {*ℓ*_1_, *ℓ*_2_, *ℓ*_*n*_, … } with minimum running cost *c*_*i*_ and remove from open

**  for all** *ℓ*_*i*_, *ℓ*_*j*_, *q*_*m*_ ∈ *T* ∣*ℓ*_*i*_ = *ℓ*_*n*_ **do**
**   **
*c*_*j*_ = *c*_*i*_ + *a*_*ij*_ (*q*_*m*_) + *h*_*j*_ (*q*_*m*_)
**   if** $c_{j}<upper$ **and** *c*_*i*_ + *a*_*ij*_ (*q*_*m*_) < *c*_*m*_ (*ℓ*_*j*_) **then**
**    **Add {*ℓ*_1_, *ℓ*_2_, *ℓ*_*i*_, *ℓ*_*j*_, … } to open

**    **
*c*_*m*_ (*ℓ*_*j*_) = *c*_*i*_ + *a*_*ij*_ (*q*_*m*_)
**   end if**
**  end for**
**  if** *ℓ*_*j*_ = *ℓ*_*f*_ **then**
**   **
*c*_*f*_ = *c*_*j*_
**   **

$upper=c_{j}$

**  end if**
** end while**

The planner tracks the current minimum cost *c*_*m*_ (initial cost set to *∞*) to reach each location (supercell) *ℓ* in the workspace within matrix *M* which contains tuples of form {*ℓ*, *c*_*m*_}. When extending the candidate sequences, the planner first selects the sequence with minimum running cost *c*_*i*_ and removes it from the set open ; it then considers the actual cost of the next transition *a*_*ij*_ as well as a monotone estimate of the future (long-term) cost *h*_*j*_ for reaching the goal state *ℓ*_*f*_ from *ℓ*_*j*_, for each *ℓ*_*j*_ reachable from *ℓ*_*i*_, with *h*_*j*_ computed in a similar way as *a*_*ij*_—see Section 5.2 for a concrete example of how *a*_*ij*_ and *h*_*j*_ can be defined with the help of a navigation function. (We note that there is considerable freedom in defining the cost functions for *a*_*ij*_ and *h*_*j*_, and it would make sense for those structures to be determined based on the desired optimization criteria for a given task.) These candidate extensions to the minimum cost sequence are evaluated using their running cost calculated as *c*_*j*_ = *c*_*i*_ + *a*_*ij*_ (*ℓ*_*j*_) + *h*_*j*_ (*ℓ*_*j*_). Candidate sequences which satisfy two conditions: (i) that $c_{j}<upper$ , and that (ii) *a*_*ij*_ + *h*_*j*_ < *c*_*m*_, where *c*_*m*_ is the current minimum cost to reach location *ℓ*_*j*_, are added to open . When the planner finds a sequence that reaches the goal state so that *ℓ*_*j*_ = *ℓ*_*f*_, it compares the cost for that sequence, *c*_*f*_, to upper . If $c_{f}<upper$ , then that sequence of transitions becomes the current minimum cost sequence to reach the goal and *c*_*f*_ is set as the new upper . This process continues until there are no more sequences in open and the best transition sequence with minimum cost *c*_*f*_ has been determined.

One note of caution is that the planner is not expected to monotonically decrease this “distance to goal surrogate” since a myopic and dynamics-agnostic gradient descent (despite any navigation function properties) offers no guarantee of task completion. A sufficiently *forward-looking* search algorithm, involving a combination of a running cost *a*_*ij*_ and a future cost *h*_*j*_ for each sequenced location *ℓ*_*j*_ is able to overcome a local cost increase along a path to the goal. An important by-product of the search for this path is the sequence of cells that mark the transitions between the activities in the solution sequence. This information is subsequently used to parameterize the underlying control laws associated with each activity, and thus implement the derived plan within the complete hybrid system.

## 5 Case study 1: a tethered UAV–UGV system

This section illustrates a slightly more complex instantiation of the planning problem shown in Figure 1. Again, we have a ugv and a uav with the capability of attaching an actuated tether between them. The goal for the system is to move the ugv from a location on one side of a vertical wall, to one on the other side. The new instantiation here includes an additional obstacle over the vertical wall (as if the wall has a window that the robots can go through). In addition, the uav has now an overhead perching location, which serves as a secure anchoring point for the tethered ugv, to lower itself down to the ground by unwinding its tether. In this problem setup, there are several interesting challenges: among them, the nonlinear continuous dynamics of the two vehicles, which approximately capture the behavior exhibited when the ugv swings in a pendulum fashion suspended by its tether and when it moves up or down from an elevated position; the obstacles that need to be avoided; the need to manage and account for the shape of the flexible tether within the cluttered workspace; and methods to identify the workspace-tether relative configurations that enable tension forces to be applied to the ugv in the desired directions.

The ugv hybrid automaton has a label set that includes the subset *A*_*g*_ = {*a*, *d*, *a*′, *r*′, *f*′} where *a* (respectively, *a*′) expresses a (respectively, tethered) rolling behavior and *d* denotes latching of the tether end on the uav, while *r*′ refers to controlled vertical ascent/descent using the tether with its other end suspended at an elevated position and *f*′ corresponds to a transient swinging motion while tethered until momentum is absorbed by impact with a workspace boundary. The uav has the a label set that includes the subset *A*_*a*_ = {*δ*, *β*, *γ*′} where *δ* enbales the uav to fly to an aerial waypoint and hover there, *β* is the landing action where the uav comes to rest on one of the workspace boundaries and *γ*′ express the tethered uav perching at its goal location to provide an anchoring point for the free end of the ugv tether. In this implementation, we allow for the ugv to *swing* (possibly impacting workspace boundaries) as it reels up or down with its tether and model this behavior in the ugv continuous dynamics.

The continuous dynamics of the combined uav-ugv system, imagined to evolve on a vertical plane for simplicity, are defined on a 10-dimensional state space *X* comprised of tuples of the form $\left\{x,y,\phi ,l,\theta \right\}$ and their first derivatives. The variable tuple (*x*, *y*, *ϕ*) parameterizes the SE(2) pose of the uav on the vertical plane of motion, and the pair (*l*, *θ*) (length/angle) provide a polar parameterization of the Cartesian position of the ugv on the same plane, relative to a *hinge point* (see Section 5.1) where the other end of the tether is attached to—during untethered horizontal motion, a virtual hinge point can be introduced on the semiaxis of ugv motion direction.

The hybrid system modeling formalism adopted (Definition 3) includes a set *W* of continuous communication variables, which for this particular case study can be realized as the set $\left\{q_{h},u,u_{1},u_{2}\right\}$ comprised of uav control inputs *u*_1_ and *u*_2_, ugv wheel/winch acceleration *u* (depending on the hybrid mode), and the (piecewise constant) location *q*_*h*_ of the hinge point relative to which the ugv position parameterization is derived.

The set of events *E* represents the edges within the join automaton graph as well as the conditions on the state variables which license each transition. In addition to the set of events themselves, it is necessary to construct functions for the state reset (jump) and guard conditions for each transition within the hybrid automaton. The event set *E* for the uav/ugv system is written as:

$$\begin{array}{llll} E_{1} & =\left\langle 1,\delta ,G_{\delta },J_{\left(1,9\right)},9\right\rangle & E_{2} & =\left\langle 9,\beta ,G_{\beta },J_{\left(9,3\right)},3\right\rangle \\ E_{3} & =\left\langle 3,\delta ,G_{\delta },J_{\left(3,9\right)},9\right\rangle & E_{4} & =\left\langle 4,\gamma^{\prime },G_{\gamma^{\prime }},J_{\left(4,5\right)},5\right\rangle \\ E_{5} & =\left\langle 1,a,G_{a},J_{\left(1,2\right)},2\right\rangle & E_{6} & =\left\langle 3,a,G_{a},J_{\left(3,2\right)},2\right\rangle \\ E_{7} & =\left\langle 3,d,G_{d},J_{\left(3,4\right)},4\right\rangle & E_{8} & =\left\langle 5,r^{\prime },G_{r^{\prime }},J_{\left(5,6\right)},6\right\rangle \\ E_{9} & =\left\langle 5,f^{\prime },G_{f^{\prime }},J_{\left(5,7\right)},7\right\rangle & E_{10} & =\left\langle 5,a^{\prime },G_{a^{\prime }},J_{\left(5,10\right)},10\right\rangle \\ E_{11} & =\left\langle 6,a^{\prime },G_{a^{\prime }},J_{\left(6,10\right)},10\right\rangle & E_{12} & =\left\langle 6,f^{\prime },G_{f^{\prime }},J_{\left(6,7\right)},7\right\rangle \\ E_{13} & =\left\langle 7,a^{\prime },G_{a^{\prime }},J_{\left(7,10\right)},10\right\rangle & E_{14} & =\left\langle 7,r^{\prime },G_{r^{\prime }},J_{\left(7,6\right)},6\right\rangle \\ E_{15} & =\left\langle 7,f^{\prime },G_{f^{\prime }},J_{\left(7,7\right)},7\right\rangle & E_{16} & =\left\langle 10,f^{\prime },G_{f^{\prime }},J_{\left(10,7\right)},7\right\rangle \\ E_{17} & =\left\langle 10,r^{\prime },G_{r^{\prime }},J_{\left(10,6\right)},6\right\rangle & E_{18} & =\left\langle 3,⋉,G_{⋉},J_{\left(3,8\right)},8\right\rangle \\ E_{19} & =\left\langle 5,⋉,G_{⋉},J_{\left(5,8\right)},8\right\rangle & E_{20} & =\left\langle 2,⋉,G_{⋉},J_{\left(2,8\right)},8\right\rangle \\ E_{21} & =\left\langle 6,⋉,G_{⋉},J_{\left(6,8\right)},8\right\rangle & E_{22} & =\left\langle 7,⋉,G_{⋉},J_{\left(7,8\right)},8\right\rangle \\ E_{23} & =\left\langle 10,⋉,G_{⋉},J_{\left(10,8\right)},8\right\rangle & \end{array}$$

The set of guards within the join automaton is constructed as constraints on the set of state variables and also the communication variables, especially in regards to collaborative activities. All velocities must be equal to zero before any transition can be initiated in the automaton. This is a condition that arises from the turn-based nature imposed on the join hybrid automaton. Guards for both vehicles are parameterized by values of their corresponding navigation function at their current Cartesian position as compared to boundary constants with *τ* → 1 and *ϵ* → 0. Additionally, the ugv guards are parameterized by function *U* (*x*, *y*) which acts as a measure of actuator authority for the vehicle. This function contains ugv mass *m*, the gravitational vector $\vec{g}$ and the gradient of the navigation function ∇*φ*:

$$\left\|m\;\vec{g}\times \frac{\nabla \varphi }{\left\|\nabla \varphi \right\|}\right\|≜U\left(x,y\right)<u_{\max}$$

Depending on the value of *U* (*x*, *y*) at a particular location, the ugv will either be able to move along the workspace boundary using its primary locomotion or it must use the tether winch to pull itself upwards. In the guards for all movement actions excluding *f*′, the action can only be initiated when when the ugv is in contact with the workspace boundary so that *φ*_*a*_ (*x*, *y*) < *τ*. For all actions which require the tether, the uav must be perched at the anchoring point so that $q_{a}=q_{a}^{f}$ . For landing action *β* to be enabled, the uav must be away for the workspace boundary so that *φ*_*a*_ (*x*, *y*) < *τ*. The opposite is true for latching action *γ*′ where the uav always starts on the boundary with *φ*_*a*_ (*x*, *y*) ≥ *τ*. Additionally, for landing action *β* to occur, the uav should be aligned with the x-position of the ugv *x*_*g*_ with this position calculated from the communication variables as $x_{g}=q_{h}^{x}+l\cos\theta$ . Finally, the value of the uav and ugv navigation function must be less than *ϵ* in order to reach the terminal location via ⋉. Thus the set of guards is written as:

$$\begin{array}{ll} G_{\delta } & =\left\{\left(x,y,\phi \right)\in X_{a}|\dot{x}=\dot{y}=\dot{\phi }=0\right\}\\ G_{\beta } & =\left\{\left(x,y,\phi \right)\in X_{a}|\dot{x}=\dot{y}=\dot{\phi }=0,\;\varphi_{a}\left(x,y\right)<\tau ,\|x-x_{g}\|<\epsilon \right\}\\ G_{\gamma^{\prime }} & =\left\{\left(x,y,\phi \right)\in X_{a}|\dot{x}=\dot{y}=\dot{\phi }=0,\;\varphi_{a}\left(x,y\right)\geq \tau \right\}\\ G_{a} & =\left\{\left(l,\theta \right)\in X_{g}|\dot{l}=\dot{\theta }=0,\;\varphi_{g}\left(x_{g},y_{g}\right)\geq \tau ,\;|u|<U\left(x_{g},y_{g}\right)\right\}\\ G_{a^{\prime }} & =\left\{\left(l,\theta \right)\in X_{g}|\dot{l}=\dot{\theta }=0,\;\varphi_{g}\left(x_{g},y_{g}\right)\geq \tau ,\;|u|\right.\\ & \;\left.<U\left(x_{g},y_{g}\right),\;q_{a}=q_{a}^{f}\right\}\\ G_{d} & =\left\{\left(l,\theta \right)\in X_{g}|\dot{l}=\dot{\theta }=0,\;\varphi_{g}\left(x_{g},y_{g}\right)\geq \tau ,\;|u|<U\left(x_{g},y_{g}\right),\;\right.\\ & \;\left.\|x-l\cos\theta \|<\epsilon ,\;\|y-l\sin\theta \|<\epsilon \right\}\\ G_{r^{\prime }} & =\left\{\left(l,\theta \right)\in X_{g}|\dot{l}=\dot{\theta }=0,\;\varphi_{g}\left(x_{g},y_{g}\right)\right.\\ & \;\left.\geq \tau ,\;|u|\geq U\left(x_{g},y_{g}\right),\;q_{a}=q_{a}^{f}\right\}\\ G_{f^{\prime }} & =\left\{\left(l,\theta \right)\in X_{g}|\dot{l}=\dot{\theta }=0,\;|u|\geq U\left(x_{g},y_{g}\right),\;q_{a}=q_{a}^{f}\right\}\\ G_{⋉} & =\left\{\left(x,y,\phi \right)\in X_{a},\left(l,\theta \right)\in X_{g}|\dot{x}\right.\\ & \;=\dot{y}=\dot{\phi }=\dot{l}=\dot{\theta }=0,x=x^{f},y=y^{f},\\ & \;\left.\varphi_{a}\left(x,y\right)<\left(1-\tau \right),\varphi_{g}\left(x_{g},y_{g}\right)<\epsilon \right\} \end{array}$$

For the tethered case study, the reset conditions which form the set of jumps takes on two forms. All uav transitions and terminating transitions admit the trivial reset where none of the state variables change in value. However, for most of the ugv transitions, the resets are utilized to reparameterize the ugv state varables according to the currently active hinge point. More details of these hinge points are covered in Section 5.1. Assume that the currently active hinge point is $q_{h}^{c}$ and the current state of the ugv is denoted by the tuple (*l*_*c*_, *θ*_*c*_). Any transition which invokes a change in the active hinge point will also result in a jump in the value of the state variables. For actions involving the tether, the hinge points denote the points in the workspace from which the tether will appear to be suspended. Actions such as *a*′ admit a virtual hinge point where the transition to the next action occurs and according to which the dynamics are parameterized. Similarly, for action *d*, the hinge points will become the location of the uav which has landed in preparation for latching where the ugv must attach the tether. If the next point in the ordered sequence of hinge points is denoted $q_{h}^{c+1}=\left(x_{h}^{c+1},y_{h}^{c+1}\right)$ and the current ugv location is denoted *q*_*c*_ = (*l*_*c*_ sin *θ*_*c*_, − *l*_*c*_ cos *θ*_*c*_), then the length *l* jumps to the Euclidean distance $d\left(q_{h}^{c+1},q_{c}\right)$ between the ugv and the new hinge point. These same variables are also utilized to reset the angle *θ* relative to the new hinge point location. The aforementioned jump conditions for each of the location pairs can then be written as:

$$\begin{array}{ll} J_{\left(1,9\right)} & :\left\{\begin{array}{c} x\mapsto \left\{x_{c}\right\}\;\\ y\mapsto \left\{y_{c}\right\}\;\\ \phi \mapsto \left\{\phi_{c}\right\}\; \end{array}\right.J_{\left(1,2\right)}:\left\{\begin{array}{c} q_{h}\mapsto q_{h}^{c+1}\;\\ l\mapsto \left\{d\left(q_{h}^{c+1},q_{c}\right)\right\}\;\\ \theta \mapsto \left\{\arctan\left(\frac{l_{c}\sin\theta_{c}-y_{h}^{c+1}}{l_{c}\cos\theta_{c}-x_{h}^{c+1}}\right)\right\}\; \end{array}\right.\\ J_{\left(2,8\right)} & :\left\{\begin{array}{c} q_{h}\mapsto \left\{q_{h}^{c}\right\}\;\\ l\mapsto \left\{l_{c}\right\}\;\\ \theta \mapsto \left\{\theta_{c}\right\},\; \end{array}\right.\; \end{array}$$

$$\begin{array}{ll} J_{\left(1,9\right)} & =J_{\left(9,3\right)}=J_{\left(3,9\right)}=J_{\left(4,5\right)}=J_{\left(3,8\right)}=J_{\left(5,8\right)}\\ J_{\left(1,2\right)} & =J_{\left(3,2\right)}=J_{\left(3,4\right)}=J_{\left(5,6\right)}=J_{\left(5,7\right)}=J_{\left(5,10\right)}=J_{\left(6,10\right)}\\ & =J_{\left(6,7\right)}=J_{\left(7,10\right)}=J_{\left(7,6\right)}=J_{\left(7,7\right)}=J_{\left(10,7\right)}=J_{\left(10,6\right)}\\ J_{\left(2,8\right)} & =J_{\left(6,8\right)}=J_{\left(7,8\right)}=J_{\left(10,8\right)} \end{array}$$

The set of invariant spaces for each location Inv is constructed in a similar fashion to the set of guards. In the initial state Location 1, the system is at rest and all state variables are given an initial value which parameterizes the initial positions of the two vehicles. Similarly, the system will remain at rest in the goal state marked by Location 8, the ugv will be at its goal coordinates and the value of the navigation function for both agents will be close to zero so that *φ*(*x*, *y*) < *ϵ*. The ugv axis is locked in Locations 4, 6, and 10, and it will not undergo angular motion so that $\dot{\theta }$ = 0. For Location 7, there is navigation function constraint *φ*_*g*_ (*x*_*g*_, *y*_*g*_) < *τ* which ensures that the ugv only remains in this location until contact is made with a workspace boundary. In all locations where the ugv is in motion, a transition and associated reset on the continuous state must occur if *l* ≤ 0. The uav will only undergo vertical motion in Location 3 so the value of *x* remains constant and *ϕ* will always equal zero. Additionally, Location 3 and Location 9 share the condition that the uav only remains in that location while it is away from the boundary of the workspace so that *φ*_*a*_ (*x*, *y*) < *τ*. The same stipulation holds for Location 5 with the added condition that the uav has not reached the goal location so that *φ*_*a*_ (*x*, *y*) ≥*ϵ*. Writing these conditions into a set of mathematical constraints, the invariant set is written as:

$$\begin{array}{ll} {Inv}_{\left(1\right)} & =\left\{\left(x,y,\phi \right)\in X_{a},\left(l,\theta \right)\in X_{g}|\dot{x}=\dot{y}=\dot{\phi }=\dot{l}=\dot{\theta }=0,\;\right.\\ & \;\left.x=x^{i},y=y^{i},l=l^{i},\phi =0,\theta =0\right\}\\ {Inv}_{\left(2\right)} & =\left\{\left(l,\theta \right)\in X_{g}|\varphi_{g}\left(x_{g},y_{g}\right)\geq \epsilon ,\;|u^{g}|<U\left(x_{g},y_{g}\right),l>0\right\}\\ {Inv}_{\left(3\right)} & =\left\{\left(x,y,\phi \right)\in X_{a}|\dot{x}=\phi =0,\;\varphi_{a}\left(x,y\right)<\tau \right\}\\ {Inv}_{\left(4\right)} & =\left\{\left(l,\theta \right)\in X_{g}|\dot{\theta }=0,\;\varphi_{g}\left(x_{g},y_{g}\right)\geq \tau ,\;l>0\right\}\\ {Inv}_{\left(5\right)} & =\left\{\left(x,y,\phi \right)\in X_{a}|\epsilon \leq \varphi_{a}\left(x,y\right)<\tau \right\}\\ {Inv}_{\left(6\right)} & =\left\{\left(l,\theta \right)\in X_{g}|\dot{\theta }=0,\;|u^{g}|>U\left(x_{g},y_{g}\right),l>0\right\}\\ {Inv}_{\left(7\right)} & =\left\{\left(l,\theta \right)\in X_{g}|\varphi_{g}\left(x_{g},y_{g}\right)<\tau ,\;|u^{g}|\geq U\left(x_{g},y_{g}\right),l>0\right\}\\ {Inv}_{\left(8\right)} & =\left\{\left(x,y,\phi \right)\in X_{a},\left(l,\theta \right)\in X_{g}|\dot{x}=\dot{y}=\dot{\phi }=\dot{l}=\dot{\theta }=0,\;\right.\\ & \;\left.\varphi_{a}\left(x,y\right)<\epsilon ,\varphi_{g}\left(x_{g},y_{g}\right)<\epsilon ,x_{g}=x_{g}^{f},y_{g}=y_{g}^{f}\right\}\\ {Inv}_{\left(9\right)} & =\left\{\left(x,y,\phi \right)\in X_{a}|\varphi_{a}\left(x,y\right)<\tau \right\}\\ {Inv}_{\left(10\right)} & =\left\{\left(l,\theta \right)\in X_{g}|\dot{\theta }=0,\;\varphi_{g}\left(x_{g},y_{g}\right)\right.\\ & \;\left.\geq \tau ,\;|u^{g}|<U\left(x_{g},y_{g}\right),l>0\right\} \end{array}$$

**FIGURE 5:**
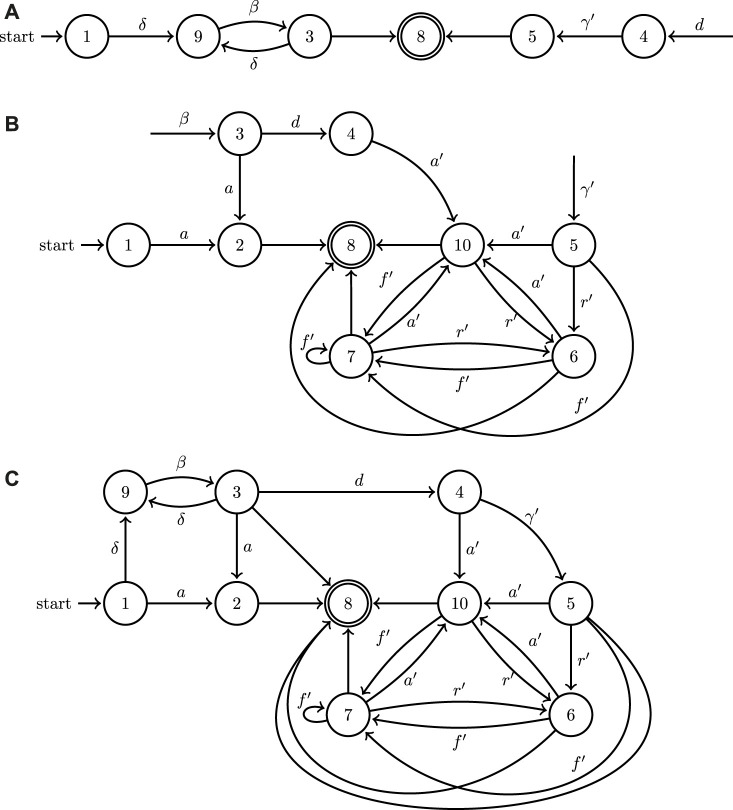
A minimal example of the application of the join operation to the directed graphs associated to the two hybrid automata modeling the UAV **(A)** and UGV **(B)** systems depicted in Figure 1, as well as the outcome of their join operation **(C)**.

The continuous dynamics (vector fields), and their assignment to corresponding locations (with reference to Figure 5) can be succinctly described as follows:

$$\begin{array}{ll} Act\left(2\right) & =\left\{\begin{array}{c} \ddot{l}=l{\dot{\theta }}^{2}-g\cos\left(\theta \right)+\frac{u}{m}\;\\ \ddot{\theta }=-\frac{2\dot{l}\dot{\theta }}{l}+\frac{g\sin\theta }{l}+N\; \end{array}\right.\\ Act\left(5,9\right) & =\left\{\begin{array}{c} \ddot{x}=-\frac{u_{1}}{m}\sin\phi \;\\ \ddot{y}=\frac{u_{1}}{m}\cos\phi -g\;\\ \ddot{\phi }=u_{2}\; \end{array}\right.Act\left(3\right)=\left\{\begin{array}{c} \ddot{x}=\ddot{\phi }=0\;\\ \ddot{y}=\frac{u_{1}}{m}\cos\phi -g\; \end{array}\right.\\ Act\left(6\right) & =\left\{\begin{array}{c} \ddot{l}=-g\cos\theta +T-\dot{l}\delta \left(t\right)+\frac{u}{m}\;\\ \ddot{\theta }=-\frac{g}{l}\sin\theta -\frac{2\dot{l}\dot{\theta }}{l}-l\dot{\theta }\delta \left(t\right)\; \end{array}\right.\\ Act\left(4\right) & =\left\{\begin{array}{c} \ddot{x}=\ddot{y}=\ddot{\phi }=\ddot{\theta }=0\;\\ \ddot{l}=\frac{u}{m}\; \end{array}\right.Act\left(10\right)=\left\{\begin{array}{c} \ddot{l}=\frac{u}{m}\;\\ \ddot{\theta }=0\; \end{array}\right.\\ Act\left(1,8\right) & =\left\{\begin{array}{c} \ddot{x}=\ddot{y}=\ddot{\phi }=\ddot{l}=\ddot{\theta }=0.\; \end{array}\right. \end{array}$$

In these equations *N* denotes the ground reaction force to the ugv, *T* is the tether tension, *m* is the ugv mass and *g* is the constant of gravitational acceleration. Location 2 corresponds to the ugv moving horizontally under traction force *u* and also allows for the robot to free-fall after driving off the edge of a surface until it impacts another workspace boundary. Similarly, Location 10 corresponds to the ugv moving horizontally under traction force *u* while tethered—the ugv is unable to free-fall assuming there is no excessive slack in the tether. Location 4 has the same dynamics for the ugv but in addition has the uav stationary which is necessary during the process of ugv latching its tether to the uav. Location 6 involves vertical displacement with horizontal swinging and also includes the possibility of collisions with workspace boundaries. Location 3 corresponds to uav vertical motion (take-off/landing), while Locations 5 and 9 describe general uav flight between waypoints and the perching of the uav on a surface. The latter two locations have identical underlying dynamics and are only differentiated based on the action’s end objective. Finally, Locations 1 and 8 mark the initial and final state of the system where both vehicles are stationary. Note that when the ugv swings suspended by the tether, its motion can be modeled as that of a pendulum with varying length and damping terms that capture the effect of possible inelastic collisions with workspace boundary, the latter activated through the action of a Dirac function *δ*(*t*).

The concrete dynamics control loops are closed as follows. For the movement of the ugv toward a selected location (hinge point), driving the input force *u* via a simple pd on the error on state *l* typically suffices. The control of the uav is realized via a differential flatness trajectory tracking control law, on uav inputs *u*_1_ and *u*_2_. For this trajectory tracking law, the collision-free (*x*, *y*) reference trajectory in the differentially flat space is constructed via the diffeomorphic transformation referenced in the following subsection.

### 5.1 Determining hinge points

The ugv dynamics are parameterized by tether *hinge points*, the location of which is critical for determining what type of motion the ugv undergoes. Hinge points denote locations on the edges of obstacles from which the ground vehicle can swing in a quasi-pendulum motion. Where these hinge points are placed depends on how the tether has been threaded through the workspace and how it deforms under the tension applied by the mass of the suspended ugv. Whether the tether can provide the necessary tension to support the weight of the vehicle, in turn, depends on its shape and relative configuration with respect to the workspace boundaries. While determining the precise configuration of a highly deformable, distributed parameter system such as a cable, chain, or tether unnecessarily complicates the planning problem and falls beyond the scope of this paper, an approximate yet realistic representation of possible tether configurations as a function of the motion history of the tethered vehicles is still necessary. One method of tether deformation approximation has been described in more detail by Sebok and Tanner (2019).

There are, in fact, infinitely many conceivable configurations for a flexible tether to weave among an arrangement of static obstacles. Yet, this multitude of configurations falls into a finite set of relevant *homotopy* classes (Bhattacharya et al., 2010; Kim et al., 2014), depending on the workspace geometry. Paths in each homotopy class can be diffeomorphically mapped onto each other. Our planning algorithm for this case study systematically enumerates those homotopy classes, and utilizes an iterative algorithm to select a valid tether path primitive (class representative) while eliminating, for instance, extensively long paths and those that tangle or wrap the tether around obstacles.

**FIGURE 6:**
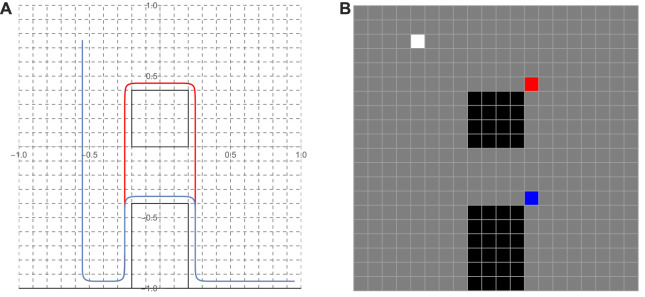
**(A)** Two representatives (red and blue) of homotopy tether curves realized as Bezier curves used as primitives; **(B)** The (color coded) hinge points associated with each of the tether primitives and the white cell marking the anchor point common to both.

The collection of hinge point locations forms a set *Q*_*h*_. In order to determine the location of these hinge points, the tether primitive identified for each homotopy class is deformed so that it tightly conforms to the edges of the obstacles in the workspace. A Bezier spline curve is then constructed using the points of the tether primitive (Figure 6). This 2D B-spline curve can be expressed in terms of its two Cartesian components *x*_*c*_(*t*) and *y*_*c*_(*t*), parameterized by *t* ∈ [0, 1]. Here we assume that the (uav) end of the tether has been anchored at point (*x*_*a*_, *y*_*a*_). This location is contained in the set of hinge points *Q*_*h*_ for every possible tether configuration. The remaining hinge points are situated where the tether is suspended as determined by the geometry of an obstacle, and these locations can be identified as the points of maximum curvature along the deformed tether curve. Hinge points are thus those pairs $\left(x_{h}\left(t\right),y_{h}\left(t\right)\right)$ corresponding to values of *t* ∈ [0, 1] that locally satisfy.

$$\underset{t\in \left[0,1\right]}{\max}\frac{d^{2}x_{c}\left(t\right)}{dt^{2}}\cdot \frac{d^{2}y_{c}\left(t\right)}{dt^{2}}\;\text{subject to}\;\left\{\begin{array}{cc} \frac{d^{2}x_{c}\left(t\right)}{dt^{2}}<0>\frac{d^{2}y_{c}\left(t\right)}{dt^{2}},\; & x_{h}>x_{a}\\ \frac{d^{2}x_{c}\left(t\right)}{dt^{2}}>0>\frac{d^{2}y_{c}\left(t\right)}{dt^{2}},\; & x_{h}<x_{a}. \end{array}\right.\;$$

The collection of all the pairs $\left(x_{h}\left(t\right),y_{h}\left(t\right)\right)$ for the locally maximizing values of *t* ∈ [0, 1] forms a finite set *Q*_*t*_ ⊂ *Q*_*h*_. Including the anchor point, the complete set of hinge points for a given homotopy class is written as *Q*_*h*_ = *Q*_*t*_ ∪ {(*x*_*a*_, *y*_*a*_)}.

### 5.2 Application: search for an action plan

The planner that searches for an (appropriately parameterized) turn-based sequence of labels that mark an action plan for the robots to take to complete the assigned task. This sequence consists of licensed transitions between supercells of the discretized workspace of the join hybrid system. An analysis of the guards and invariants of the join hybrid automaton for this system leads to the definition of 13 different supercells (out of 400 individual cells—see Figure 6), with each supercell associated to a unique system activity, and 19 possible transitions between those supercells, as licensed by the join hybrid automaton.

The planner weighs the different options for possible transitions out of a given supercell based on cost functions evaluated on continuous state space variables of the join hybrid system. To facilitate computation, this continuous domain is quantized using the centroids of the discretized workspace cells. Several possibilities exist for cost functions to guide the planner’s search; a good trade-off between greedy and longer-range planning is offered by a combination of a short-term with a long-term cost function. To see how the planner’s assessment is carried out in this particular case study, assume that the system is currently at cell *ℓ*_*i*_ and considers transitioning to some cell *ℓ*_*j*_ which belongs to a different supercell than the one it is currently in. Now assume that navigation function *φ* is available on the continuous state space of the join hybrid automaton, assuming its minimum at the region where the system task specification is satisfied, and being uniformly maximum at the system’s workspace boundary. With some abuse of notation, we will write *φ*(*ℓ*_*i*_) to express the value of the navigation function at the centroid of the transition cell *ℓ*_*i*_ that the system currently occupies, and similarly denote *φ*(*ℓ*_*j*_) as the corresponding value at the cell currently being assessed. (Note that *φ*(*ℓ*_*f*_) ≡ 0) With *D* (*ℓ*_*i*_, *ℓ*_*j*_) denoting the Euclidean distance between the centroids of cell *i* and *j*, the local and future cost functions that quantify the cost-benefit ratio of a transition between 2 cells are given as

$$a_{ij}=\frac{D\left(\ell_{i},\ell_{j}\right)}{\left|\varphi \left(\ell_{i}\right)-\varphi \left(\ell_{j}\right)\right|}\;\;\;\;h_{j}=\frac{D\left(\ell_{j},\ell_{f}\right)}{\varphi \left(\ell_{j}\right)},$$

respectively. In this particular implementation, the action-specific cost of initiating uav motion is set at a value higher than the maximum cost of a ugv action, to reflect the fact that uav motion is energetically way more “expensive” compared to ugv motion. This setting practically ensures that the uav will be deployed only if the ugv cannot move where it needs to go on its own.

**FIGURE 7:**
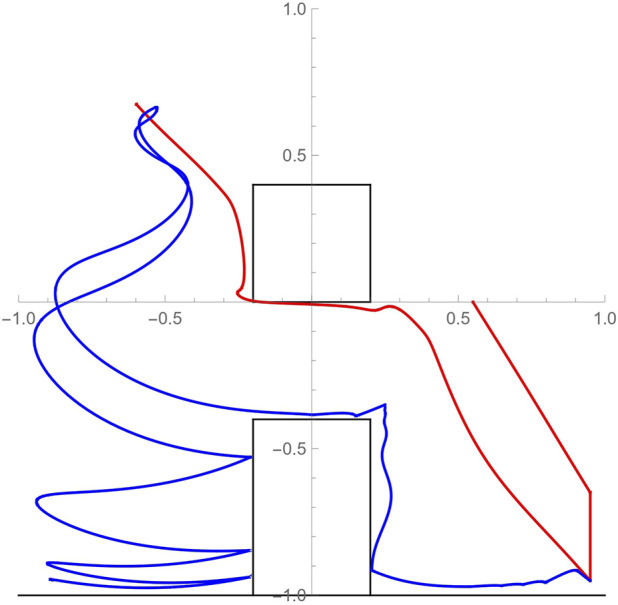
Simulated paths of the uav (red) and ugv (blue) motion through the constrained workspace as they implement the cooperative action plan. The motion path of the uav from an initial hovering position at (0.55, 0) to its perching configuration (−0.55, 0.75) consists of three distinct segments: reaching waypoint (0.95, − 0.65) directly above designated landing spot; landing; and tracking a reference trajectory to the perching location avoiding the overhead obstacle. The motion path of the ugv consists of the following segments: swinging motion to the left from initial location (0.95, − 0.95) until initial impact at the foot of the vertical wall after the uav has perched; tethered-assisted ascent on the side of the wall; a swinging ascending motion from the top of wall to the uav’s perching point; and finally, a swinging descent to the desired position with periodic collisions on the left side of the wall.

After comparing label sequences, the planner settles on *δ β d γ*′ *f*′ *f*′. This plan involves the activity where the uav reaches a waypoint (via *δ*) and subsequently lands (via *β*) at a designated rendezvous location for tether latching; the ugv subsequently moves in to latch its tether on the uav (via *d*); the uav then lifts off and goes to perch at a designated elevated spot (via *γ*′); the ugv ascends to the first hinge point by reeling in the tether and swinging until it impacts the wall (via *f*′); and finally, the ugv ascends to the anchoring location and reels out the tether to descend towards the goal location (via *f*′). The reason why *f*′ appears repeated is because the associated activity is parameterized differently each time: in the former case, the hinge point is at the upper rightmost corner of the vertical wall obstacle while the ugv ascends, whereas in the latter case the hinge point switches to the perched position of the uav and the ugv performs controlled ascent/descent while suspended from the uav’s perched position. This is an specific example of why a naive, purely discrete search over the join hybrid automaton graph representation is very likely to miss the desired solutions. A numerical implementation of this plan is showcased in Figure 7. The motion paths of the two vehicles are color coded.

### 5.3 Extension to a heterogeneous system with three agents

In principle, and absent computational considerations, the proposed methodology is subject to no limitation on the number of collaborative agents. In fact, it is modular in the sense that new agents can be added subsequently. The planning methodology can leverage the added capability as long as the existing agents are modeled to interact with the new modalities. To illustrate this, and without explicit analytical description for the sake of brevity, consider the above uav/ugv system with an additional agent consisting of a common robotic manipulator. This robotic manipulator is located near the goal position for the ugv and can grasp some object located to the goal location and deposit it into a container on the ugv.

**FIGURE 8:**
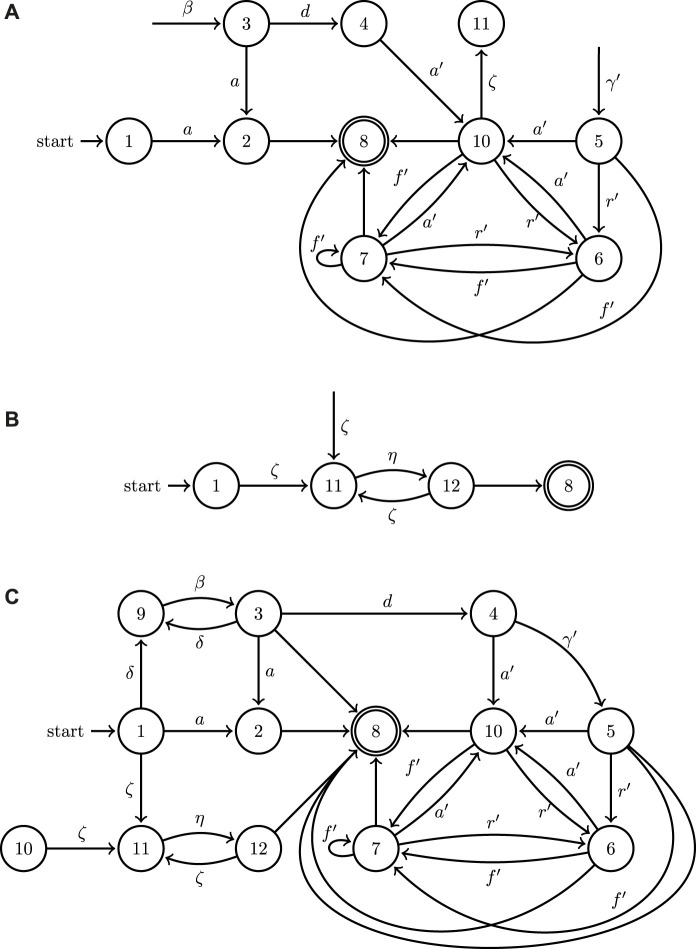
An example of the extension of the join operation to the directed graphs associated with the three hybrid automata for an updated model of the UAV **(A)**, a model of a robotic manipulator **(B)**, and UGV model (Figure 5A), as well as the outcome of their join operation **(C)**.

The robotic manipulator label set *A*_*r*_ would consist of two actions: *ζ* represents picking up an object and *η* represents placing an object in a new location. In this new system, the uav automaton from Figure 5A remains unchanged. The ugv is updated as shown in Figure 8A to reflect that action *ζ* can occur after ugv action *a*′. Taking the *join* of the hybrid automaton graph for the robotic manipulator (Figure 8B) with the automata for the uav and ugv, the join hybrid automaton is produced with the discrete components shown in Figure 8C. This join hybrid automaton for the extended three agent system contains both the individual functionality of the three agents as well as their collaborative possibilities.

### 5.4 Comparison to possible alternative methodologies

A couple of alternative methodologies were also employed to solve the cooperative planning problem of this section, neither of which produced a valid solution to the planning problem. The first one is based on a hybrid system optimal control design framework, available for systems with dynamics in polynomial form (Zhao et al., 2020). The second was a more general multi-robot motion and task planner called grstaps (Messing et al., 2022). These two solvers represent approaches at the two ends of the methodological spectrum, with the optimal control formulation putting more emphasis on the continuous dynamics, and grstaps primarily leveraging abstraction and discretization to scale up.

It was found that both approaches had difficulty addressing this problem, with challenges seeming to stem from dynamics nonlinearities. The major limitation of the hybrid optimal control approach is that it can only generate solutions for hybrid systems containing polynomial dynamics. This becomes a critical issue when attempting to generate meaningful planning comparisons for the uav/ugv system as the nonlinear dynamics of this system are not adequately represented in polynomial form approximations. This resulted in this planning approach only being able to generate plans for a simplified system utilizing only the ugv. The hybrid optimal control approach was incapable of producing an action sequence and control outputs when the specified goal required use of the swinging action *f*′.

On the other hand, grstaps required a particularly fine workspace discretization to be able to reasonably keep track of the nontrivial nonlinear system dynamics. The potential upside of this planner is its ability to efficiently perform task planning, scheduling, and motion planning. However, the primarily discrete nature of this algorithm limits the planner’s ability to account for the dynamic restrictions which are essential to constructing a feasible action sequence.

In conclusion, none of the aforementioned alternative methodologies were capable of leveraging the unique new behaviors that are made possible through physical interaction amongst the component systems when only presented with the individual robot dynamics (as in Figure 3). In fact, even when the outcome of the join operation was *fed directly* as input to these planners, they still struggled: the optimal control algorithm could not produce meaningful sequences of inputs for the uav and ugv while grstaps only produced valid cooperative plans when, *in addition* to the join automaton, it was also explicitly presented with the same workspace partition and discretization utilized above. The poor performance of these two planners on the uav/ugv system meant that it was impossible to create a meaningful comparison between the existing planners and the methodology outlined within this work.

## 6 Case study 2: the dual UGV system

The previous section illustrated in some detail how the proposed planning methodology can be applied to coordinate a heterogeneous robot team comprised of a uav that can tether itself to a ugv. This section aims to reinforce the point that the presented methods are not tailored to a particular multi-robot system or a mechanism of physical interaction between agents. While it is true that it may not always be possible to formulate any arbitrary multi-robot planning problem into one that fits the proposed framework, in principle the methodology is applicable to a reasonably wide class of small-scale heterogeneous multi-robot systems.

**FIGURE 9:**
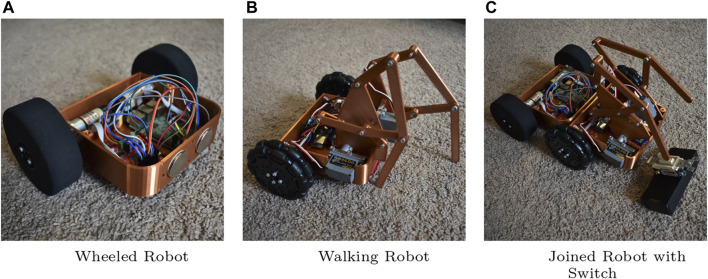
Another heterogeneous system consisting of **(A)** a wheeled robot and **(B)** a walking robot. Using magnets embedded in the robot frames, the vehicles can join together to interact with objects such as a light switch **(C)**. A video demonstrating a sequence involving the two ugvs joining and then moving together can be found at: http://research.me.udel.edu/ btanner/videos.html.

**FIGURE 10:**
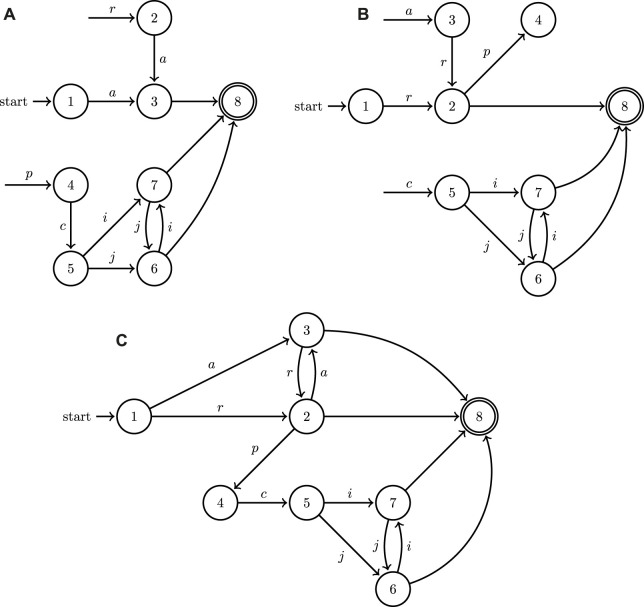
A minimal example of the application of the join operation to the directed graphs representing two hybrid automata with the walking ugv **(A)**, wheeled ugv **(B)** of Figure 9 as well as the outcome of their join operation **(C)**.

To this end, this section considers a different heterogeneous multi-robot configuration consisting of two ugvs. Here the ugvs are heterogeneous due their different locomotion modalities and motion degrees of freedom. The first robot (Figure 9A; Figure 10) is a (nonholonomic) wheeled robot with a differential drive. The second robot (Figure 9B) is a walking mechanism that utilizes a Klann linkage mechanism to locomote, pulling two unactuated wheels in the back for balance. This second robot has only one degree of freedom (it cannot turn); it does, however, have a second actuator capable of tilting its legs along a vertical plane when somehow supported on its other side. This external support can be realized by magnets located on the front of the rolling robot and the rear of the walking robot. Note that neither of the agents individually can manipulate the environment, but when connected by means of the magnets (Figure 9C), the raised legs become end effectors and the contraption takes the form of a basic two degree of freedom (dof) manipulator. This allows the joined robot to perform other tasks such as flipping a switch or pressing a button.

The planning approach of the previous sections (without the tether analysis) can be applied to this system too. For this case, assume that the task at hand is to have the robots flip a switch like the one shown in Figure 9. Here, the planning algorithm coordinates the two robots to join and form the mobile manipulator, and then steers it toward its final objective. The details of the computational implementation are omitted since the smaller number of degrees of freedom in this problem and the simplicity of the individual robot kinematics render the problem relatively straightforward compared to the case study of Section 5; the hybrid automata models and synthesis are however described at a similar level of detail as in the uav–ugv case in the following section.

### 6.1 Hybrid automata models

The walking ugv has a label set *A*_*w*_ = {*a*, *c*, *i*, *j*} where *a* expresses the basic one-dimensional walking motion, *c* is the connection action where the walking ugv joins itself to the wheeled ugv using the magnetic connection, *i* represents the interaction of the joined vehicle with the environment and *j* expresses the two-dimensional motion of the joined vehicle in the horizontal plane. Similarly, the wheeled ugv has label set *A*_*r*_ = {*r*, *p*, *i*, *j*} with *r* being the basic two-dimensional motion action in the horizontal plane, *p* being an action which positions the wheeled ugv behind the walking ugv in prepartion for connection and *i*, *j* being the same as in the walking ugv. Note that *i* and *j* are labels for both component hybrid systems; however, the associated activities can only be triggered after the individual robots have joined into a single vehicle.

The continuous dynamics of this dual ugv system are defined within a 5th dimensional state space *X* composed of tuples of the form $\left\{\phi_{w},\theta_{w},x_{r},y_{r},\theta_{r}\right\}$ . The Cartesian coordinate system is established so that that that the origin is positioned at the initial position of the walking ugv and the *x*-axis is aligned with its orientation. Thus, the wheeled ugv’s initial position is parameterized relative to the initial position of the walking ugv. In this case, (*x*_*r*_, *y*_*r*_, *θ*_*r*_) parameterize the SE(2) pose of the wheeled ugv on the plane of motion while the pair (*ϕ*_*w*_, *θ*_*w*_) parameterize both the Cartesian position of the walking ugv along the *x*-axis as well as the rotational position and inclination of the 2 dof manipulator when the agents are joined together. Specifically, *ϕ*_*w*_ tracks the net rotations of the Klann linkage while *θ*_*w*_ provides its inclination angle relative to the ground. When the robots are detached and the walking motion is enabled, *θ*_*w*_ will remain constant so that *θ*_*w*_ = 0.

For the dual ugv system, the set *W* of continuous communication variables $\left\{q_{i},u_{1}^{w},u_{2}^{w},u_{1}^{r},u_{2}^{r}\right\}$ . comprised of walking ugv control inputs $u_{1}^{w}$ and $u_{2}^{w}$ , wheeled ugv control inputs $u_{1}^{r}$ and $u_{2}^{r}$ , and the (piecewise constant) location *q*_*i*_ of the current position within the plane where the joined robot must interact with its environment.

The event set *E* representing all transitions in the graph of the join automaton can be written as:

$$\begin{array}{llll} E_{1} & =\langle 1,a,G_{a},J_{\left(1,3\right)},3\rangle & E_{2} & =\langle 2,a,G_{a},J_{\left(2,3\right)},3\rangle \\ E_{3} & =\langle 4,c,G_{c},J_{\left(4,5\right)},5\rangle & E_{4} & =\langle 5,j,G_{j},J_{\left(5,6\right)},6\rangle \\ E_{5} & =\langle 5,i,G_{i},J_{\left(5,7\right)},7\rangle & E_{6} & =\langle 7,j,G_{j},J_{\left(7,6\right)},6\rangle \\ E_{7} & =\langle 6,i,G_{i},J_{\left(6,7\right)},7\rangle & E_{8} & =\langle 1,r,G_{r},J_{\left(1,2\right)},2\rangle \\ E_{9} & =\langle 3,r,G_{r},J_{\left(3,2\right)},2\rangle & E_{10} & =\langle 2,p,G_{p},J_{\left(2,4\right)},4\rangle \\ E_{11} & =\langle 3,⋉,G_{⋉},J_{\left(3,8\right)},8\rangle & E_{12} & =\langle 6,⋉,G_{⋉},J_{\left(6,8\right)},8\rangle \\ E_{13} & =\langle 7,⋉,G_{⋉},J_{\left(7,8\right)},8\rangle & E_{14} & =\langle 2,⋉,G_{⋉},J_{\left(2,8\right)},8\rangle \end{array}$$

Again, the key constructions within the set of events are the set of guards and resets (jumps) for each transition. Under the assumption that the system will reach a steady state before the next action, the Klann mechanism must be at rest for an action to be triggered which is written ${\dot{\phi }}_{w}=0$ for the guard of each action. This assumption also implies that all velocities must equal zero for the wheeled ugv before any transition can occur. For actions that require the Klann linkage for locomotion, the mechanism must be in contact with the ground so that *θ*_*w*_ = 0. The position of the walking ugv along the *x*-axis can be determined by the conversion $x_{w}=s\frac{\phi_{w}}{\pi }$ where *s* is the stride length of the Klann linkage. This conversion allows for comparisons to determine where the walking ugv is positioned to the wheeled ugv. For movement action *a*, the walking ugv must not be in the same location as the wheeled ugv. For the positioning action *p* to occur, *y*_*r*_ = 0 to ensure that the robot is along the horizontal *x*-axis where the walking ugv is positioned. For connection action *c* to occur, the wheeled ugv must be properly positioned on the *x*-axis and aligned with the walking ugv magnets. For joint actions *i* and *j*, the Klann linkage must be raised to not interfere with movement so that *θ*_*w*_ > 0. Also, for the joined vehicle to interact with an object with *i*, the vehicle position (measured using the wheeled ugv coordinates *q*_*r*_) must be sufficiently close to the interaction location *q*_*i*_. Finally, the system must be at its goal position and at rest for the terminating transition ⋉ to be enabled. With these conditions in mind, the guards of the join automaton are written as follows:

$$\begin{array}{ll} G_{a} & =\left\{\left(\phi_{w},\theta_{w}\right)\in X_{w}|{\dot{\phi }}_{w}=\theta_{w}=0,x_{r}\neq s\frac{\phi_{w}}{\pi }\;\|\;y_{r}\neq 0\right\}\\ G_{c} & =\left\{\left(\phi_{w},\theta_{w}\right)\in X_{w}|{\dot{\phi }}_{w}=\theta_{w}=0,\theta_{r}=0,y_{r}=0,x_{r}\neq s\frac{\phi_{w}}{\pi }\right\}\\ G_{r} & =\left\{\left(x_{r},y_{r},\theta_{r}\right)\in X_{r}|{\dot{x}}_{r}={\dot{y}}_{r}={\dot{\theta }}_{r}=0\right\}\\ G_{p} & =\left\{\left(x_{r},y_{r},\theta_{r}\right)\in X_{r}|{\dot{x}}_{r}={\dot{y}}_{r}={\dot{\theta }}_{r}=0,y_{r}=0\right\}\\ G_{i} & =\left\{\left(x_{r},y_{r},\theta_{r}\right)\in X_{r}|{\dot{\phi }}_{w}=\theta_{w}={\dot{x}}_{r}={\dot{y}}_{r}={\dot{\theta }}_{r}\right.\\ & \left.=0,\;\theta_{w}>0,\;\|q_{r}-q_{i}\|<\epsilon \right\}\\ G_{j} & =\left\{\left(x_{r},y_{r},\theta_{r}\right)\in X_{r}|{\dot{\phi }}_{w}=\theta_{w}={\dot{x}}_{r}={\dot{y}}_{r}={\dot{\theta }}_{r}=0,\;\theta_{w}>0\right\}\\ G_{⋉} & =\left\{\left(\phi_{w},\theta_{w}\right)\in X_{w},\left(x_{r},y_{r},\theta_{r}\right)\in X_{r}|{\dot{\phi }}_{w}\right.\\ & ={\dot{\theta }}_{w}={\dot{x}}_{r}={\dot{y}}_{r}={\dot{\theta }}_{r}=0,\\ x_{r} & \left.=x_{r}^{f},y_{r}=y_{r}^{f},\theta_{r}=\theta_{r}^{f}\right\} \end{array}$$

For the dual ugv case study, there are no resets on the state variables which allows every transition to admit the trivial jump condition:

$$J_{\left(\ell_{i},\ell_{j}\right)}:\left\{\begin{array}{c} \phi_{w}\mapsto \left\{\phi_{wc}\right\}\;\\ \theta_{w}\mapsto \left\{\theta_{wc}\right\}\;\\ x_{r}\mapsto \left\{x_{rc}\right\}\;\\ y_{r}\mapsto \left\{y_{rc}\right\}\;\\ \theta_{r}\mapsto \left\{\theta_{rc}\right\}\; \end{array}\right.\forall \ell_{i},\ell_{j}\in L$$

The set of invariant spaces for each location Inv(*ℓ*) is constructed in a similar fashion to the set of guards. While the guards parameterize conditions on the state and communication variables for a particular transition to occur, the invariant spaces are solely functions of the state variables and represent conditions for the automaton execution to remain within the location reached by its associated transition label. In the initial state Location 1, the system is at rest, all velocities are zero and walking ugv state variables are initialized to zero. The wheeled ugv state variables are initialized to some initial value relative to the initial location of the walking ugv. Additionally, the system will also remain at rest in the goal state marked by Location 8. While the wheeled ugvis within Location 2 and Location 6, the derivative of one of the state variables must be non-zero so that the vehicle remains in motion. Additionally for the wheeled ugv to remain in Location 6, the position of the vehicle must not be located at the desired interaction location. Within Location 4, the wheeled vehicle is only allowed to rotate so that it always has angular velocity but zero linear velocities. While the walking ugv is within Location 3, the inclination angle of the mechanism *θ*_*w*_ remains fixed at 0 and the angular velocity of the arm rotation must take on some non-zero value. In Locations 5 and 7, either the angular velocity of the linkage or of the mechanism rotation must maintain a non-zero value. Additionally, in Locations 6 and 7, the arm mechanism is lifted to accommodated the joined vehicle so that *θ*_*w*_ > 0 within those locations. Finally, within Location 7, the joined vehicle is sufficiently close to the prescribed interaction location and velocities for the wheeled ugv variables are zero as all movement will be performed by the walking ugv portion of the vehicle. Taking all of these conditions into account, the invariant set is written as:

$$\begin{array}{ll} {Inv}_{\left(1\right)} & =\left\{\left(\phi_{w},\theta_{w}\right)\in X_{w},\left(x_{r},y_{r},\theta_{r}\right)\in X_{r}|{\dot{\phi }}_{w}\right.\\ & ={\dot{\theta }}_{w}={\dot{x}}_{r}={\dot{y}}_{r}={\dot{\theta }}_{r}=0,\;\\ \phi_{w} & \left.=\theta_{w}=0,x_{r}=x_{r}^{i},y_{r}=y_{r}^{i},\theta_{r}=\theta_{r}^{i}\right\}\\ {Inv}_{\left(2\right)} & =\left\{\left(x_{r},y_{r},\theta_{r}\right)\in X_{r}|{\dot{x}}_{r}>0\;\|\;{\dot{y}}_{r}>0\;\|\;{\dot{\theta }}_{r}>0\right\}\\ {Inv}_{\left(3\right)} & =\left\{\left(\phi_{w},\theta_{w}\right)\in X_{w}|\theta_{w}=0,\;|{\dot{\phi }}_{w}|>0\right\}\\ {Inv}_{\left(4\right)} & =\left\{\left(x_{r},y_{r},\theta \right)\in X_{r}|{\dot{x}}_{r}={\dot{y}}_{r}=0,\;{\dot{\theta }}_{r}>0\right\}\\ {Inv}_{\left(5\right)} & =\left\{\left(\phi_{w},\theta_{w}\right)\in X_{w}||{\dot{\phi }}_{w}|>0\;\|\;|{\dot{\theta }}_{w}|>0\right\}\\ {Inv}_{\left(6\right)} & =\left\{\left(\phi_{w},\theta_{w}\right)\in X_{w},\left(x_{r},y_{r}\right)\in X_{r}|{\dot{\theta }}_{w}=0,\;\theta_{w}>0,\;\right.\\ & \left.{\dot{x}}_{r}>0\;\|\;{\dot{y}}_{r}>0\;\|\;{\dot{\theta }}_{r}>0,\;q_{r}\neq q_{i}\right\}\\ {Inv}_{\left(7\right)} & =\left\{\left(\phi_{w},\theta_{w}\right)\in X_{w},\left(x_{r},y_{r},\theta_{r}\right)\in X_{r}|{\dot{x}}_{r}={\dot{y}}_{r}={\dot{\theta }}_{r}=0,\;\right.\\ & \left.|{\dot{\phi }}_{w}|>0\;\|\;|{\dot{\theta }}_{w}|>0,\;\theta_{w}>0,\;\|q_{r}-q_{i}\|<\epsilon \right\}\\ {Inv}_{\left(8\right)} & =\left\{\left(\phi_{w},\theta_{w}\right)\in X_{w},\left(x_{r},y_{r},\theta_{r}\right)\in X_{r}|{\dot{\phi }}_{w}\right.\\ & \left.={\dot{\theta }}_{w}={\dot{x}}_{r}={\dot{y}}_{r}={\dot{\theta }}_{r}=0\right\} \end{array}$$

The activities (continuous vector fields) for the two ugv hybrid systems can be expressed as follows:

$$\begin{array}{llll} Act\left(1,8\right) & =\left\{\begin{array}{c} {\dot{x}}_{r}={\dot{y}}_{r}={\dot{\theta }}_{r}={\dot{\phi }}_{w}={\dot{\theta }}_{w}=0\; \end{array}\right. & Act\left(5\right) & =\left\{\begin{array}{c} {\dot{\phi }}_{w}=u_{1}^{w}\;\\ {\dot{\theta }}_{w}=u_{2}^{w}\; \end{array}\right.\\ Act\left(2\right) & =\left\{\begin{array}{c} {\dot{x}}_{r}=u_{1}^{r}\cos\theta \;\\ {\dot{y}}_{r}=u_{1}^{r}\sin\theta \;\\ {\dot{\theta }}_{r}=u_{2}^{r}\; \end{array}\right. & Act\left(6\right) & =\left\{\begin{array}{c} {\dot{x}}_{r}=u_{1}^{r}\cos\theta \;\\ {\dot{y}}_{r}=u_{1}^{r}\sin\theta \;\\ {\dot{\theta }}_{r}=u_{2}^{r}\;\\ {\dot{\phi }}_{w}={\dot{\theta }}_{w}=0\; \end{array}\right.\\ Act\left(3\right) & =\left\{\begin{array}{c} {\dot{\phi }}_{w}=u_{1}^{w}\;\\ {\dot{\theta }}_{w}=0\; \end{array}\right. & Act\left(7\right) & =\left\{\begin{array}{c} {\dot{x}}_{r}={\dot{y}}_{r}={\dot{\theta }}_{r}=0\;\\ {\dot{\phi }}_{w}=u_{1}^{w}\;\\ {\dot{\theta }}_{w}=u_{2}^{w}\; \end{array}\right.\\ Act\left(4\right) & =\left\{\begin{array}{c} {\dot{x}}_{r}=0\;\\ {\dot{y}}_{r}=0\;\\ {\dot{\theta }}_{r}=u_{2}^{r}\; \end{array}\right. & \end{array}$$

The above equations for Act(*ℓ*) thus capture the continuous dynamics of the dual ugv system within the join hybrid automaton. We see that the wheeled ugv is modeled using standard unicycle dynamics while the walking ugv is a basic single integrator model. Note that the joined ugv inherits the unicycle dynamics of the wheeled ugv while the walking ugv inputs now control the movement of the 2-DOF manipulator. Location 2 corresponds to the wheeled ugv moving within the 2-Dimensional plane and similarly Location 3 corresponds to the walking ugv moving horizontally along the *x*-axis. Location 4 is a rotational only action of the wheeled ugv which orients the magnets on the front of the robot so that they are aligned with the magnets on the rear of the walking ugv along the *x*-axis. Location 5 corresponds to the magnetic connection between the two ugvs performed by the walking ugv, and the walking ugv also raising its legs off the ground. Location 6 represents the motion of the joined vehicle with the wheeled ugv component providing the locomotion towards interaction locations. Location 7 corresponds to the manipulator on the joined vehicle interacting with the environment while the vehicle remains stationary. Finally, locations 1 and 8 mark the initial and final state of the system where both vehicles are stationary.

### 6.2 Application: search for an action plan

The construction of an action plan for the dual ugv system proceeds in a similar fashion to the process outlined in the uav-ugv study. The A* planner weighs the possible transitions based on cost functions evaluated on continuous state space variables of the join hybrid system and the continuous domain is divided and quantized using the centroids of the resulting workspace cells. While there are still multiple valid selections for cost functions as inputs to the planner, the reduced system complexity allows for the selection of simplified cost functions without effecting planner convergence.

Suppose that the planner is given the system at cell *ℓ*_*i*_ and considers transitioning it to some cell *ℓ*_*j*_; the cell containing the goal location (here: the location of the light switch) will be denoted *ℓ*_*f*_. With *D* (*ℓ*_*i*_, *ℓ*_*j*_) expressing the Euclidean distance between the centroids of cell *i* and *j*, the short and long-term cost functions that quantify the cost-benefit ratio of a transition between 2 cells are given more simply (no workspace obstacles are considered here):

$$a_{ij}=D\left(\ell_{i},\ell_{j}\right)\;\;\;\;\;h_{j}=D\left(\ell_{j},\ell_{f}\right),$$

respectively. For this system these action-specific costs may only apply to events labeled with symbols in (*a*, *r*, *j*). For the remaining three events, namely, positioning *p*, connection *c*, and interaction *i*, fixed costs of the form

$$a_{ij}=C\;\;\;\;\;h_{j}=0,$$

suffice, given that these as generally fixed duration cooperative events. For simplicity, positioning *p* is set to take place at some fixed distance behind the current location of the walking ugv.

Comparing event sequence costs, the planner this time settles on the sequence *r p c j i*. This plan represents the wheeled ugv driving (*r*) to a location behind the walking ugv and rotating itself to align itself to connect with other vehicle (*p*); the walking ugv making the magnetic connection with the wheeled ugv and lifting the manipulator mechanism (*c*); the joined ugv driving towards interaction location *q*_*i*_ (*j*); and finally, the joined ugv interacting with a light switch at the designated location (*i*).

### 6.3 Physical implementation

Realizations of the walking robot and wheeled robot designs were achieved using a mix of 3D printed and off-the-shelf components as seen in Figure 9. Local controllers corresponding to each location were implemented using microcontrollers on each of the robotic agents. A Raspberry Pi on the wheeled robot acts as the coordinating controller in this case and sends the required actions to the walking robot controller to ensure synchronization between the two vehicles. The walking robot is controlled by an ESP32 and commands are received via a Wi-Fi connection with the Raspberry Pi. In testing, the vehicles were able to magnetically connect, move to the interaction location, and flip a standard light switch mounted within the workspace of the walking robot manipulator. In this manner, the ugvs are capable of successfully implementing the sequence calculated by the A* planner and completing the desired task.

## 7 Conclusion

Cooperative planning for tasks not feasible without physical interaction and nontrivial mechanical coupling between heterogeneous robotic agents is particularly challenging and pushes existing multi-robot planning and control methodologies to their limits. The approach outlined in this paper overcomes this challenge by (a) incorporating aspects of the underlying continuous dynamics that capture the intricacies of physical interaction between the heterogeneous robot teammates and expressing their effects through a hybrid dynamical system modeling framework; (b) subsequently abstracting these continuous dynamics into discrete modes resting on appropriately parameterized control loops, and finally and arguably more importantly, (c) adapting and introducing a novel composition operation for hybrid dynamical systems which is capable of expressing cooperative group behaviors that are neither the union nor the intersection of those of its group members. The combined heterogeneous multi-robot system planning and control architecture is capable of revealing as solutions new cooperative behaviors that were not previously achievable. Yet more work is needed to further develop and extend this approach to enable more automation in the modeling phase, allow concurrent subsystem actions, and achieve higher computational efficiency. Additionally, future work will attempt to adapt the turn-based nature of automata execution to allow for agents to perform concurrent actions. The relaxation of this restriction is likely nontrivial within the confines of the current modeling framework.

## Data Availability

The original contributions presented in the study are included in the article/Supplementary Material, further inquiries can be directed to the corresponding author.
